# Random forest-based identification and ranking of predictive factors for physical activity in Chinese college students

**DOI:** 10.3389/fpubh.2025.1699606

**Published:** 2025-11-20

**Authors:** Ding-you Zhang, Hu Lou, Jun Liu, Bo Li

**Affiliations:** Institute of Sports Science, Nantong University, Nantong, China

**Keywords:** university students, machine learning, random forest, physical activity, socio-ecological model

## Abstract

**Objective:**

To explore the key predictors of physical activity (PA) levels of Chinese university students, and to analyse the predictive roles of different variables and their relative importance by means of the Random Forest (RF) algorithm.

**Methods:**

A cross-sectional study was conducted using a stratified whole-group sampling method, covering 17 provinces of the country and collecting 10,182 valid questionnaires. Assessment of PA levels using the Physical Activity Rating Scale-3 (PARS-3) divides participants into attainment and non-attainment groups. The independent variables encompass the individual and interpersonal organisational levels of the socio-ecological model (SEM), comprising a total of 39 variables. These variables include demographic characteristics, psycho-behavioural factors, and social support, which were measured using several standardised scales. Feature importance analysis was performed using the Random Forest algorithm, and the model parameters were optimised with a grid search and 5-fold cross-validation to identify the most significant factors predicting PA.

**Results:**

The RF model had an accuracy of 0.704 and an AUC value of 0.762. Characteristic importance analysis revealed that exercise adherence (exercise behaviour), sex, exercise adherence (effort investment), mastery of sports skills, exercise motivation (ability), alcohol consumption level, exercise adherence [emotional experience, exercise motivation (social), and exercise motivation (fun) ranked as the top nine predictive factors]. Specifically, all sub-dimensions of exercise adherence (exercise behaviour) positively predict PA (SHAP values > 0); sex, males are more likely than females to meet the standard group criteria (OR > 1, *p* < 0.001); mastery of sports skills correlates positively with PA levels; and among alcohol consumption level, ‘occasional drinking’ shows a negative correlation with the standard attainment rate (*p* < 0.001).

**Conclusion:**

Exercise adherence, sex, mastery of sports skills, and alcohol consumption level are significant factors predicting PA levels among Chinese university students. Recommendations for promoting PA include enhancing the “emotional value” and social attributes of exercise, addressing female students’ willingness to participate, and improving physical capabilities through skills training to effectively elevate activity levels.

## Introduction

Physical activity (PA) refers to any bodily movement produced by skeletal muscle contraction that requires energy expenditure (1). Extensive research demonstrates that maintaining a certain level of PA can significantly alleviate negative emotional symptoms, such as depression and stress, among university students (2), preserve physical health (3), and improve health-related quality of life (2). In 2020, the World Health Organisation (WHO) issued guidelines on PA and sedentary behaviour (3). These guidelines emphasise that to mitigate the adverse health effects of sedentary behaviour, adults should engage in 150–300 min of moderate-intensity aerobic PA, or 75–150 min of vigorous-intensity aerobic PA, or an equivalent combination of both, per week. A 2024 study involving 5.7 million participants revealed that levels of insufficient PA continue to rise globally (4). Despite global recommendations, insufficient PA remains an urgent public health concern, especially among young people (5). A 2022 WHO briefing note indicates that 81% of adolescents aged 13–24 globally fail to meet recommended PA levels. This represents a 5-percentage-point increase since 2010, signalling a persistent widening of the youth PA deficit (6). A 2024 global study of 5.7 million participants revealed a continued rise in insufficient PA, particularly among individuals aged 18–24 (4). Nationwide surveys in China indicate that approximately 70–80% of university students fail to meet WHO-recommended PA levels (5, 7). Recent trends also show low PA levels among adolescents in certain regions, where only 13.2% of subjects meet WHO recommendations (8). Notably, boys’ PA levels tend to exceed those of girls (9). Furthermore, increased financial constraints and academic pressures among some university students further diminish opportunities for PA engagement (10). Given PA’s pivotal role in promoting physical and mental health during early adulthood, understanding and addressing the factors contributing to physical inactivity among Chinese university students has significant public health implications. However, current evidence on PA levels among Chinese university students remains fragmented, with most studies examining only a few variables such as self-efficacy (11), sex (12), social support (5), screen time, and stress (13). To address this gap and improve the current situation of insufficient PA among Chinese university students, this study selected a national sample of 10,182 college students from 16 provinces and municipalities across China. It simultaneously integrated these predictors of student PA to identify the most significant key variables, thereby enhancing PA levels and promoting the physical health of university students.

Bronfenbrenner (14) first systematically proposed the Social-Ecological Model (SEM). Building upon Bronfenbrenner’s social ecological theory, McLeroy (15) introduced a hierarchical analytical framework. Employing spatial metaphors, this framework categorises influencing factors across five progressively broader dimensions: individual, interpersonal, organisational, community, and policy. The individual level focuses on intrinsic personal characteristics, including health perceptions, behavioural patterns, and self-regulation capabilities. The interpersonal level emphasises supportive interactions within social networks such as family members and peer groups. The organisational level examines the structural influences exerted by institutional entities, such as schools and enterprises, through normative frameworks and resource allocation. The community level integrates social and environmental factors, such as cultural traditions and public facilities, within geographical spaces. At the policy level, as a macro-level driving system, it continuously permeates all levels through laws, regulations, cultural values, and national strategies (16, 17). As a core theoretical framework in health behaviour research, the SEM demonstrates significant advantages in analysing the determinants of individual health behaviours through its multidimensional analytical system (18). In this study, the model enables a stratified examination of factors predicting PA levels, precisely identifying root-cause factors within target groups to guide the formulation of tailored health intervention programmes. Consequently, the individual and interpersonal/organisational levels of the SEM were selected as the analytical framework. Factors potentially predicting university students’ PA levels were incorporated into this model to explore the interplay between individual and interpersonal/organisational dimensions, thereby providing systematic solutions for promoting students’ physical health. The exclusion of the community and policy levels was primarily based on the following considerations: This study constitutes the foundational phase of predictive model construction, aiming to rapidly identify key individual factors amenable to intervention. It seeks to provide low-cost, high-return intervention targets for physical education in higher education institutions. All research samples originate from internal university environments, where sporting resources, curriculum design, and management systems are constrained by unified educational policies and institutional frameworks, resulting in minimal policy-level variation. University students’ primary activity spaces are concentrated on campus, with community sporting resources exerting far less influence on their sporting behaviour than on-campus factors. McLeroy et al. (15) similarly note that the SEM does not necessitate the simultaneous inclusion of all hierarchical variables. Researchers may prioritise the most intervention-worthy levels based on study objectives and resource constraints. Overseas studies have also adopted a hierarchical modelling strategy, first establishing individual models before expanding to socio-policy models (19, 20). This research follows these pathways, demonstrating both practical and theoretical validity.

With the rapid advancement of artificial intelligence technology, machine learning applications in fields such as health management and sports science have gradually matured. In 2016, the State Council’s ‘Healthy China 2030’ Planning Outline also proposed for the first time to ‘improve the physical fitness monitoring system and develop applications for big data on national physical fitness monitoring’ (21). Although machine learning has been applied in sports education (22, 23), competitive sports (24, 25), and athletic performance prediction (26), its application in identifying predictors of PA among university students remains unexplored. Machine learning possesses advantages such as ‘massive data processing’, ‘capturing non-linear relationships’, and ‘feature importance ranking’. It can integrate multi-dimensional predictive factors to construct high-precision prediction models, providing technical support to overcome traditional research limitations and enhance the scientific rigour and practicality of physical literacy studies (27). It holds broad application prospects in the prediction and assessment of physical fitness and health. Therefore, to thoroughly explore the predictors of PA among Chinese university students and formulate targeted physical intervention policies and health promotion strategies, this study proposes to employ four machine learning models—Logistic Regression (LR), Random Forest (RF), eXtreme Gradient Boosting (XGBoost), and Light Gradient Boosting Machine (LightGBM)—to model the predictors of PA among Chinese university students. By integrating the SEM with machine learning, this approach enriches and expands analytical methods within the field of PA research, offering new perspectives for related studies.

## Methods

### Survey subjects

The survey participants comprised students enrolled in ordinary higher education institutions within mainland China, with the list of such institutions referenced from the Ministry of Education’s ‘National List of Ordinary Higher Education Institutions (as of 20 June 2024)’. Following the principles of stratified cluster sampling, representative samples were drawn from 17 provinces across the nation. The final sample comprised 155 higher education institutions, divided into three categories: 42 institutions in ‘Category I’ (provincial capital cities), 73 in ‘Category II’ (municipalities with average socio-economic conditions), and 40 in ‘Category III’ (municipalities with relatively weaker socio-economic conditions). It should be specifically noted that Nantong University, as the lead institution for the project, primarily assumes responsibilities for research design, coordination, and ethical oversight. The sampling framework for this study encompasses the entire nation, with data collection not concentrated in Nantong or its provincial jurisdiction. The nationwide sampling methodology detailed below ensures geographical diversity within the sample, effectively mitigating regional biases that might otherwise arise from a single institution’s dominance.

### Sampling method

The study primarily employed epidemiological survey methods. To ensure the national representativeness of the sample, the investigation covered most regions of China, including Jiangsu, Shanghai, Shandong, Jilin, Henan, Sichuan, Chongqing, Guizhou, Yunnan, Shaanxi, Gansu, Qinghai, Ningxia, Xinjiang, Guangxi, Inner Mongolia, and Hainan provinces. This geographical scope encompasses eastern, central, western and north-eastern China, representing diverse levels of socio-economic development. Consequently, it provides macro-level support for the subsequent conclusions’ external validity when extrapolated to the broader population of Chinese university students. A total of 10,182 valid questionnaires were obtained. The survey participants comprised undergraduate students enrolled in China’s regular higher education institutions, specifically including junior college students and undergraduates, but excluding postgraduate students (master’s and doctoral candidates). Samples were stratified by geographical region (e.g., province, municipal administrative level) and university type (e.g., comprehensive universities versus local institutions). Considering the sample size requirements for the RF model employed in this study, we assessed sample adequacy using empirical rules and the events per variable (EPV) criterion (28). RF models typically necessitate sufficient observations per category to ensure model stability, with a generally recommended minimum sample size of at least 10 times the number of independent variables. This study involved 39 independent variables, necessitating a sample size of at least 390 individuals. The final sample size (*N* = 10,182) substantially exceeded the required sample size. At the same time, the positive event count in the target group (*n* = 4,529) also met the EPV > 10 criterion, providing sufficient support for the robustness of model training and validation (28).

This study employed stratified, cluster, and multistage sampling methods to select survey subjects. The specific sampling procedures are as follows:

#### Determine the sampling location

To ensure the representativeness of the test subjects, each province (autonomous region, municipality) was allocated an average of three sampling locations. Based on drawing equal samples from different cities, the specific approach was as follows: - Prefecture-level cities under the jurisdiction of each province or autonomous region were selected as sampling locations. Among these, provincial capitals were designated as ‘Category I’ sampling locations; The selection principles for the other two prefecture-level cities are as follows: while considering the geographical distribution within the province or autonomous region, one prefecture-level city with average socio-economic development is designated as a ‘Category II’ sampling location, and one prefecture-level city with relatively underdeveloped socio-economic conditions is designated as a ‘Category III’ sampling location. In municipalities directly under the central government, sample selection does not need to adhere to the principles above, with random cluster sampling being the primary method of choice. However, the principle of three sampling locations must be observed quantitatively.

#### Determine the sampling unit

Four primary considerations guided the selection of sampling units: firstly, the higher education institution must be a fully accredited institution registered with the Ministry of Education, including higher vocational colleges; secondly, the unit must satisfy sampling requirements (namely age, student numbers, Grade distribution, etc.); thirdly, the unit must have a designated questionnaire distribution coordinator and demonstrate willingness for long-term participation in the monitoring programme; fourthly, the institution’s students must have returned for the autumn semester.

#### Grouping

Participants were divided into two groups by sex (male and female), and then further categorised into eight sample groups by Grade.

### Data cleaning rules and selection

During data preprocessing, responses exhibiting logical errors, omissions, inaccuracies, or unidentifiable entries were either retested or excluded to ensure the authenticity and validity of the data. Valid questionnaires were incorporated according to the following rules: Step 1: Exclude questionnaires where the full name of the institution was illegible; Step 2: Exclude questionnaires where the respondent’s age was recorded as under 18 or over 25; Step 3: Exclude questionnaires where at least 21 consecutive response codes were identical; Step 4: Following the above exclusions, the average completion time for remaining questionnaires was 539 s. Questionnaires with completion times falling within the ranges [0, 5%] and [95, 100%] were removed.

Ultimately, this study selected 10,182 Chinese university students aged 18–25 as research subjects. Questionnaire data collection was conducted online, with researchers and supervising lecturers present during the data acquisition process. This study was approved by the Ethics Committee of Nantong University [Approval No. Tongda Ethics (2022) 70, dated 16 February 2022]. All participants signed informed consent forms detailing the study’s objectives, methodology, potential risks, and subjects’ rights. This ensured voluntary participation based on ethical review and full disclosure. All participants were informed that the questionnaire would take approximately 12 min to complete and that they could withdraw at any time without repercussions. A pre-survey was conducted before implementation to refine the questionnaire design based on the feedback received. To minimise potential social desirability bias and recall bias inherent in self-reported data, the following measures were implemented during this study: (1) Standardised scales validated for reliability and validity among Chinese university students (e.g., PARS-3) were prioritised to ensure cultural adaptation and measurement efficacy; (2) Conducting a pre-survey prior to formal questionnaire administration, refining question phrasing based on feedback to enhance clarity and unambiguity of items; (3) Researchers or supervising tutors were present during data collection to provide standardised explanations for participants’ queries; (4) Rigorous data cleaning protocols were applied, alongside confidential processing of sample data. Nevertheless, measurement of subjective constructs remains primarily reliant on self-report, a limitation further addressed in the discussion section.

### Selection and coding of scales

#### PA levels

The PA levels of university students in this study were measured using the Physical Activity Rating Scale-3 (PARS-3), developed by Japanese scholar Hashimoto Kimio in 1990 and subsequently translated and revised by Chinese scholar Liang Deqing in 1994 (29). The PARS-3 assesses PA volume across three dimensions: intensity, frequency, and duration per session. Each dimension comprises five options, with scores ranging from 1 to 5. The total score ranges from 0 to 100 points, where a higher score indicates a greater volume of PA.


Calculation formula: PA score=Intensity×(Time-1)×Frequency

The PARS-3 normative classification for Chinese adults is as follows: low activity level (≤19 points), moderate activity level (20–42 points), and high activity level (≥43 points) (29). The PARS-3 exhibits a Cronbach’s *α* coefficient of 0.78, GFI = 0.93, AGFI = 0.92, CFI = 0.88, RMSEA = 0.06, and test–retest reliability of 0.82. The scale has undergone validation of reliability and validity within China, demonstrating sound reliability and validity, and serves well as a general research instrument. According to the World Health Organisation’s PA and Sedentary Behaviour Guidelines, adults aged 18–64 should achieve 150–300 min of moderate-intensity or 75–150 min of vigorous-intensity aerobic activity per week, or an equivalent combination (5). Accordingly, this study combined the ‘moderate activity level’ and ‘high activity level’ categories assessed by PARS-3 into a ‘compliant group’ to define PA levels meeting the guideline requirements.

#### Independent variable

This study, based on McLeroy’s hierarchical analysis framework (28), selected a total of 39 variables categorised into individual, interpersonal, and organisational levels. This approach aims to comprehensively cover key factors influencing university students’ PA at individual, interpersonal, and organisational levels. Variable selection adhered to three principles: first, theory-driven selection, ensuring all variables corresponded to constructs defined at the individual or interpersonal-organisational levels within the SEM (30); secondly, literature support, with all included variables having been demonstrated in prior research to exhibit significant associations with PA among adolescents or university students (30–33); thirdly, practical feasibility, prioritising psychological and behavioural characteristics observable and amenable to intervention within campus settings. Accordingly, variables reflecting individual intrinsic traits, cognitive patterns, and behavioural modes—such as mastery of sports skills, exercise motivation, psychological resilience, and health literacy—were explicitly categorised as individual-level factors. Variables reflecting the quality of an individual’s interactions with social networks—such as student peer relationships, family support, and socially oriented exercise motivation—were classified as interpersonal-organisational level factors. For instance, psychological resilience and its sub-dimensions are categorised as individual-level factors, as they constitute intrinsic psychological assets that enable individuals to cope with stress and challenges. This study aims to address the shortcomings of previous research, which often focused on isolated factors without a systematic framework, by employing this multi-level, multi-factor integrated analysis. This approach enables more precise identification of key targets for PA interventions.

### Introduction to machine learning models

Machine learning models possess the capacity to handle non-linear relationships and higher-order interaction effects, enabling them to effectively capture the intricate interplay mechanisms between cross-level variables within SEM (34); Secondly, they exhibit inherent tolerance to multicollinearity, rendering them suitable for the multidimensional, potentially correlated set of predictor variables in this study (35); furthermore, these models accommodate both continuous and categorical variables without stringent assumptions on variable distributions, thereby enhancing their generalisability and practical adaptability (36). More significantly, through feature importance ranking and SHAP value interpretation, these models can quantify each variable’s marginal contribution to prediction outcomes (37). This aligns logically with the SEM’s emphasis on the independent and synergistic effects of multi-level factors on behaviour, providing data-driven evidence for identifying key intervention targets. Therefore, this study selected the following four machine models for the construction of prediction models and conducted comparisons between the models.

#### LR

LR, proposed by David Cox in 1958, stands as one of the most maturely applied members within the family of generalised linear models. It employs the logit transformation to map linear combinations onto the 0–1 probability space, enabling maximum likelihood estimation for binary or multi-class classification tasks (38). Despite its simple structure, LR is widely employed in disease risk prediction, credit scoring, consumer behaviour modelling, and sports outcome forecasting due to its highly interpretable coefficients, computational efficiency, and suitability for small-to-medium sample sizes (39–41). Furthermore, its output probabilities can be directly converted into risk assessment scores, providing transparent and auditable decision-making foundations for public health policies and sports betting markets. Specific LR parameters include: C: The reciprocal of regularisation strength; smaller floating-point values impose stronger penalties, preventing overfitting. Typical search range: 0.01–100. penalty: Regularisation type, selectable as L1 (sparsity coefficient), L2 (weight decay), or elasticnet (hybrid of both); L1 suits high-dimensional sparse scenarios, while elasticnet requires additional specification of l1_ratio. solver: Optimisation algorithm, liblinear is suitable for small datasets and supports L1/L2, while saga supports elasticnet and large datasets. max_iter: Maximum iteration count, default 100; may be increased to 200–500 if model fails to converge. class_weight: Category weighting; setting to balanced enables automatic inverse weighting based on sample frequency to mitigate class imbalanc (38).

#### RF

RF, as a vital component of machine learning, constitute an ensemble learning algorithm based on decision trees, first proposed by Leo Breiman in 2001 (42). They enhance predictive accuracy and robustness by constructing multiple decision trees and aggregating their predictions through voting (43). RF employ a bootstrap aggregating (bagging) algorithm comprising multiple decision trees as predictors. Each decision tree functions as a weak learner, with the final prediction determined through voting or averaging. Existing research demonstrates that RF have achieved significant predictive outcomes in finance (44), medical fields (45–47), energy management (48, 49), and education (50). It has also been applied in sports to predict match outcomes (51, 52), and athletic performance (53). Core RF parameters include: n_estimators: Number of decision trees in the forest. Larger values within 50–500 reduce variance but increase computational load linearly. Typically, 100–300 strikes a balance between accuracy and efficiency. max_depth: The maximum depth of a single tree, controlling its fitting capability; greater depth increases overfitting risk, commonly set to 3–10. min_samples_split: The minimum number of samples required for node splitting; increasing this value suppresses overfitting; typical values range from 2 to 20. min_samples_leaf: Minimum leaf node sample size, synergistically controlling tree complexity with the above parameter; typically set to 1–5. max_leaf_nodes: Maximum leaf nodes per tree. Once set, the tree grows using ‘best-first’ expansion until the leaf node limit is reached or impurity gains plateau. Common search range [4, 6, 8, 10]. Smaller values yield simpler models and prevent overfitting. min_impurity_decrease: Minimum impurity reduction required for node splitting. Splitting ceases below this threshold. Typical grid [0, 0.01, 0.02]. Increasing this value prunes and simplifies the model, enhancing generalisation capability (42).

#### XGBoost

XGBoost, open-sourced by Tianqi Chen in 2016, is a high-performance ensemble tree method that incorporates second-order derivatives, regularisation, and sparsity-aware algorithms within the gradient boosting framework (54). It progressively optimises the objective function through additive training, fitting the negative gradient of residuals from the previous round at each iteration to achieve strong generalisation capabilities (54). Its built-in gain-based feature importance and SHAP value interpretation modules enable researchers to quantify the marginal contributions of multi-level influencing factors, providing interpretable pathways for precise interventions. n_estimators: Number of boosting rounds, i.e., the number of trees; commonly used values range from 50 to 300. max_depth: Maximum depth per tree, default 6; 3–8 suffices during training, as excessive depth risks overfitting. learning_rate: Learning rate, 0.01–0.3; lower values enhance robustness but require more trees. Subsample: Sampling proportion of training samples per round, 0.6–1.2 introduces randomness to reduce variance. Gamma: Minimum loss reduction required for node splitting; higher values make the model more conservative. Adjustable between 0 and 0.5. reg_lambda: Regularisation penalty for weights, controlling model complexity. Common combinations range from 0 to 5.

#### LightGBM

LightGBM was released by the Microsoft team in 2017, significantly reducing computational and memory overhead through its histogram-based gradient boosting algorithm (55). Employing a leaf-wise growth strategy and supporting direct input of categorical features, it achieves training speeds several to dozens of times faster while maintaining accuracy (55). LightGBM demonstrates exceptional performance in scenarios involving extremely large samples and high-dimensional sparse data (56), When integrated with the SHAP interpretability framework, the model clearly reveals the interactive effects of different match scenarios or environmental-level variables on outcomes. This provides rapid, scalable, and interpretable evidence support for both sports tactics and public health policy. num_leaves: Number of leaf nodes per tree, jointly controlling complexity with max_depth; typically set to 0.5–1 times 2^(max_depth). max_depth: Maximum tree depth, recommended at 3–8 for leaf-wise growth mode. learning_rate (eta): Same as XGBoost, commonly set at 0.05–0.2. colsample_bytree: Sample and feature sampling ratio, introducing randomness at 0.6–1.0. min_child_samples: Minimum number of samples per leaf node, with higher values suppressing overfitting; commonly set at 10–100.

### Statistical methods and machine learning model construction

In this study, data processing primarily utilised three software packages: SPSS 27.0, Excel, and DAMAS. The entire process can be divided into several key stages: (1) Preliminary processing of data collected via the Wenshu Xing platform was conducted using Excel, including re-measurement or removal of incomplete or anomalous data. (2) Data analysis was conducted on the collected student data. For continuous variables, the chi-square test was employed to investigate differences in PA levels. The η^2^ value ranged between 0 and 1. According to Cohen’s d standards, an effect size of 0.01 indicates a small effect, 0.06 denotes a moderate effect, and 0.14 signifies a large effect (57). A larger η^2^ suggests a stronger association between the continuous variable and PA levels, as well as a greater explanatory power for differences in PA levels. Conversely, a smaller η^2^ signifies a weaker association and reduced explanatory power. For categorical variables, cross-tabulation was employed to examine the strength of associations between sex, Grade, age, and student location regarding exercise, romantic involvement, and depression. Cramer’s V ranges from 0 to 1, with higher values indicating a stronger association between categorical variables. Values of 0.0 ≤ V < 0.1 correspond to no or extremely weak association, 0.1 ≤ V < 0.3 to weak association, 0.3 ≤ V < 0.5 to moderate association, and V ≥ 0.5 to strong association (58). (3) Four machine learning models—LR, RF, XGBoost, and LightGBM—were constructed to predict the influencing factors of university students’ PA levels and rank these factors. The training set comprised 80% of the data, with the remaining 20% allocated to the test set. Model parameters were optimised through cross-validation of each parameter combination within a predefined parameter space, with the highest accuracy on the validation set serving as the optimization objective. The parameter combination demonstrating optimal generalisation capability and stability on the test set was ultimately selected to determine the model’s optimal parameter values (59, 60). (4) Following determination of optimal model parameters, performance evaluation utilised confusion matrices and their derived metrics: accuracy, precision, recall, and F1 score. Each confusion matrix metric ranges from 0 to 1, with higher values indicating superior model performance. Additionally, AUC (Area Under the ROC Curve) was employed to quantify overall model performance. AUC denotes the area under the ROC curve, ranging from 0 to 1, where higher values indicate superior model performance. (5) The optimal model is selected, and SHAP (SHapley Additive exPlanations) values are employed to interpret the model’s predictions, providing directional and magnitude information regarding feature contributions.

## Results

### Descriptive analysis

Table 1 results indicate that among 10,182 Chinese university students aged 18–25, the attainment rate for the standardised group was 44.5%, with males (63.8%) significantly higher than females (28.9%); higher Grades (third and fourth years at approximately 57%) outperformed lower Grades (first and second years at approximately 43%). Mastery of sports skills exhibited a dose–response effect: those proficient in ≥2 skills achieved 52.4% compliance, compared to only 22.6% among those proficient in 0 skills. For exercise motivation, individuals who ‘strongly agreed’ with all five dimensions (‘ability, fun, social, health, appearance’) demonstrated compliance rates of 60–70%, significantly higher than those who were ‘neutral’ (30–37%). Regarding health behaviours, those who occasionally drank alcohol or smoked achieved higher compliance rates, whereas daily screen time exceeding 8 h was associated with reduced compliance. Psychologically, the compliant group exhibited greater psychological resilience, self-efficacy, and life satisfaction, alongside lower depression levels. Scores across all three dimensions of health literacy were significantly superior to those of the non-compliant group.

**Table 1:** Descriptive analysis summary.

Variable		PA				
		Non-compliant group (5653)		Compliant group (4529)		Effect size
Categorical variable						
Sex n/%	Male	1,647	36.2	2,900	63.8	x^2^ **=** 1238.989 Cramer’s V = 0.349 *p* < 0.001
	Female	4,006	71.1	1,629	28.9	
Grade n/%	Freshman year	3,400	56.2	2,652	43.8	x^2^ = 60.711 Cramer’s V = 0.077 *p* < 0.001
	Sophomore year	1862	57.7	1,367	42.3	
	Third year	275	43.4	358	56.6	
	Senior year	116	43.3	152	56.7	
Mastery of sports skills n/%	0 items	489	77.4	143	22.6	x^2^ = 511.079 Cramer’s V = 0.224 *p* < 0.001
	1 Items	1963	69.4	867	30.6	
	2 Items and above	3,201	47.6	3,519	52.4	
Screen time n/%	≤3 h/d	551	48.3	590	51.7	x^2^ = 27.783 Cramer’s V = 0.052 *p* < 0.001
	3 h/d less than or equal to screen time≤8 h/d	4,135	56.6	3,167	43.4	
	>8 h/d	967	55.6	772	44.4	
Exercise motivation (Health) n/%	Not at all	11	34.4	21	65.6	x^2^ = 468.573 Cramer’s V = 0.215 *p* < 0.001
	Not quite	98	60.5	64	39.5	
	Not clear	1,464	65.2	780	34.8	
	Comparatively	3,308	59.3	2,274	40.7	
	Exactly	772	35.7	1,390	64.3	
Exercise motivation (Appearance) n/%	Not at all	18	36.0	32	64.0	x^2^ = 405.576 Cramer’s V = 0.200 *p* < 0.001
	Not quite	153	56.5	118	43.5	
	Not clear	1,582	62.6	945	37.4	
	Comparatively	3,128	59.9	2096	40.1	
	Exactly	772	36.6	1,338	63.4	
Exercise motivation (Fun) n/%	Not at all	18	43.9	23	56.1	x^2^ = 584.112 Cramer’s V = 0.240 *p* < 0.001
	Not quite	130	71.4	52	28.6	
	Not clear	1,556	66.8	774	33.2	
	Comparatively	3,222	58.9	2,247	41.1	
	Exactly	727	33.7	1,433	66.3	
Exercise motivation (Ability) n/%	Not at all	23	54.8	19	45.2	x^2^ = 826.822 Cramer’s V = 0.285 *p* < 0.001
	Not quite	214	72.8	80	27.2	
	Not clear	1953	70.0	836	30.0	
	Comparatively	2,870	56.9	2,178	43.1	
	Exactly	593	29.5	1,416	70.5	
Exercise motivation (Social) n/%	Not at all	58	49.2	60	50.8	x^2^ = 578.544 Cramer’s V = 0.238 *p* < 0.001
	Not quite	381	64.0	214	36.0	
	Not clear	2,250	64.2	1,256	35.8	
	Comparatively	2,465	57.4	1826	42.6	
	Exactly	499	29.8	1,173	70.2	
Nearsightedness status n/%	Near-sighted	4,627	58.6	3,274	41.4	x^2^ = 132.216 Cramer’s V = 0.114 *p* < 0.001
	Not near-sighted	1,026	45.0	1,255	55.0	
Average monthly living expenses n/%	1,000 yuan and below	654	58.9	457	41.1	x^2^ = 41.291 Cramer’s V = 0.064 *p* < 0.001
	1,000 to 2,000 yuan	4,407	56.1	3,453	43.9	
	2000 to 3,000 yuan;	520	50.9	501	49.1	
	3,000 to 4,000 yuan	45	43.3	59	56.7	
	more than 4,000 yuan	27	31.4	59	68.6	
Self-rated health level n/%	Poor	107	63.3	62	36.7	x^2^ = 290.724 Cramer’s V = 0.169 *p* < 0.001
	Fair	1,691	63.1	987	36.9	
	Good	2,110	60.4	1,381	39.6	
	Very good	1,244	48.5	1,322	51.5	
	Excellent	501	39.2	777	60.8	
Sleep quality n/%	Very good	1,425	52.6	1,286	47.4	x^2^ = 41.764 Cramer’s V = 0.064 *p* < 0.001
	Not bad	2,864	58.4	2044	41.6	
	Fair	1,178	54.6	979	45.4	
	Very poor	186	45.8	220	54.2	
Self-esteem level n/%	Very inconsistent	61	49.2	63	50.8	x^2^ = 133.541 Cramer’s V = 0.115 *p* < 0.001
	Inconsistent	887	60.5	578	39.5	
	Consistent	3,787	58.0	2,745	42.0	
	Very consistent	918	44.5	1,143	55.5	
Student peer relationships n/%	Not at all	141	53.8	121	46.2	x^2^ = 153.769 Cramer’s V = 0.123 *p* < 0.001
	Not quite	1,166	60.9	750	39.1	
	Comparatively	3,440	58.0	2,492	42.0	
	Exactly	906	43.7	1,166	56.3	
Smoking behaviour n/%	Never smoked	5,285	58.2	3,789	41.8	x^2^ = 255.142 Cramer’s V = 0.158 *p* < 0.001
	Occasional smoker	163	34.2	314	65.8	
	Smoker but not addicted	124	35.3	227	64.7	
	Addicted but controlled	33	24.8	100	75.2	
	Addicted but not controlled	48	32.7	99	67.3	
Relationship status n/%	Single	3,343	61.5	2094	38.5	x^2^ = 169.449 Cramer’s V = 0.129 *p* < 0.001
	In a relationship	1,316	49.4	1,348	50.6	
	Passionately in love	994	47.8	1,087	52.2	
Mobile phone addiction tendency n/%	Not at all	386	41.9	536	58.1	x^2^ = 133.392 Cramer’s V = 0.114 *p* < 0.001
	Not quite	1,241	55.0	1,017	45.0	
	Not clear	1888	59.9	1,263	40.1	
	Comparatively	1807	57.9	1,312	42.1	
	Exactly	331	45.2	401	54.8	
Alcohol consumption level n/%	Never drink	2,899	64.0	1,632	36.0	x^2^ = 268.181 Cramer’s V = 0.162 *p* < 0.001
	Occasional drinker	2032	50.2	2012	49.8	
	Drinker but not addicted to alcohol	531	48.4	567	51.6	
	Addicted to alcohol but in control	96	39.0	150	61.0	
	Addicted to alcohol but not in control	89	37.2	150	62.8	
	Has an addiction and drinks daily	6	25.0	18	75.0	

Continuous variable					F	η^2^	*p*
Psychological resilience (goal focus) M/sd	18.518	3.279	19.227	3.657	106.105	0.010	<0.001
Psychological resilience (emotional control) M/sd	17.232	3.847	16.417	4.067	107.383	0.010	<0.001
Psychological resilience (positive cognition) M/sd	15.268	2.601	15.762	2.945	80.611	0.008	<0.001
Psychological resilience (family support) M/sd	17.777	2.415	17.766	2.691	0.048	<0.001	0.826
Psychological resilience (interpersonal assistance) M/sd	17.454	3.077	17.278	3.362	7.620	0.001	0.006
Psychological resilience level M/sd	86.250	9.483	86.450	10.472	1.020	<0.001	0.313
Exercise adherence (exercise behaviour) M/sd	50.417	7.550	56.307	8.813	1317.810	0.115	<0.001
Exercise adherence (level of effort investment) M/sd	9.551	1.429	9.795	1.593	66.233	0.006	<0.001
Exercise adherence (emotional experience) M/sd	9.048	1.556	9.363	1.752	91.957	0.009	<0.001
Exercise adherence level M/sd	9.340	1.408	9.752	1.571	193.773	0.019	<0.001
Health literacy (healthcare) M/sd	27.938	3.938	28.909	4.463	135.579	0.013	<0.001
Health literacy (disease prevention and control) M/sd	13.546	2.602	15.866	2.884	1814.827	0.151	<0.001
Health literacy (health promotion) M/sd	18.117	2.996	20.132	3.403	1006.937	0.090	<0.001
Health literacy level M/sd	18.754	2.875	20.309	3.258	653.115	0.060	<0.001
Depression level M/sd	15.377	4.763	15.145	5.105	5.588	0.001	0.018
Anxiety level M/sd	10.674	3.996	10.550	4.309	2.243	<0.001	0.134
General self-efficacy M/sd	18.008	5.095	19.608	5.681	223.702	0.022	<0.001
Self-emotional management ability M/sd	32.382	9.458	32.242	10.954	0.478	<0.001	0.489
Life satisfaction level M/sd	23.149	5.453	23.891	6.076	42.042	0.004	<0.001
Leisure guilt level M/sd	29.875	4.239	30.808	4.790	108.534	0.011	<0.001

### Model performance metrics

**Figure 1:**
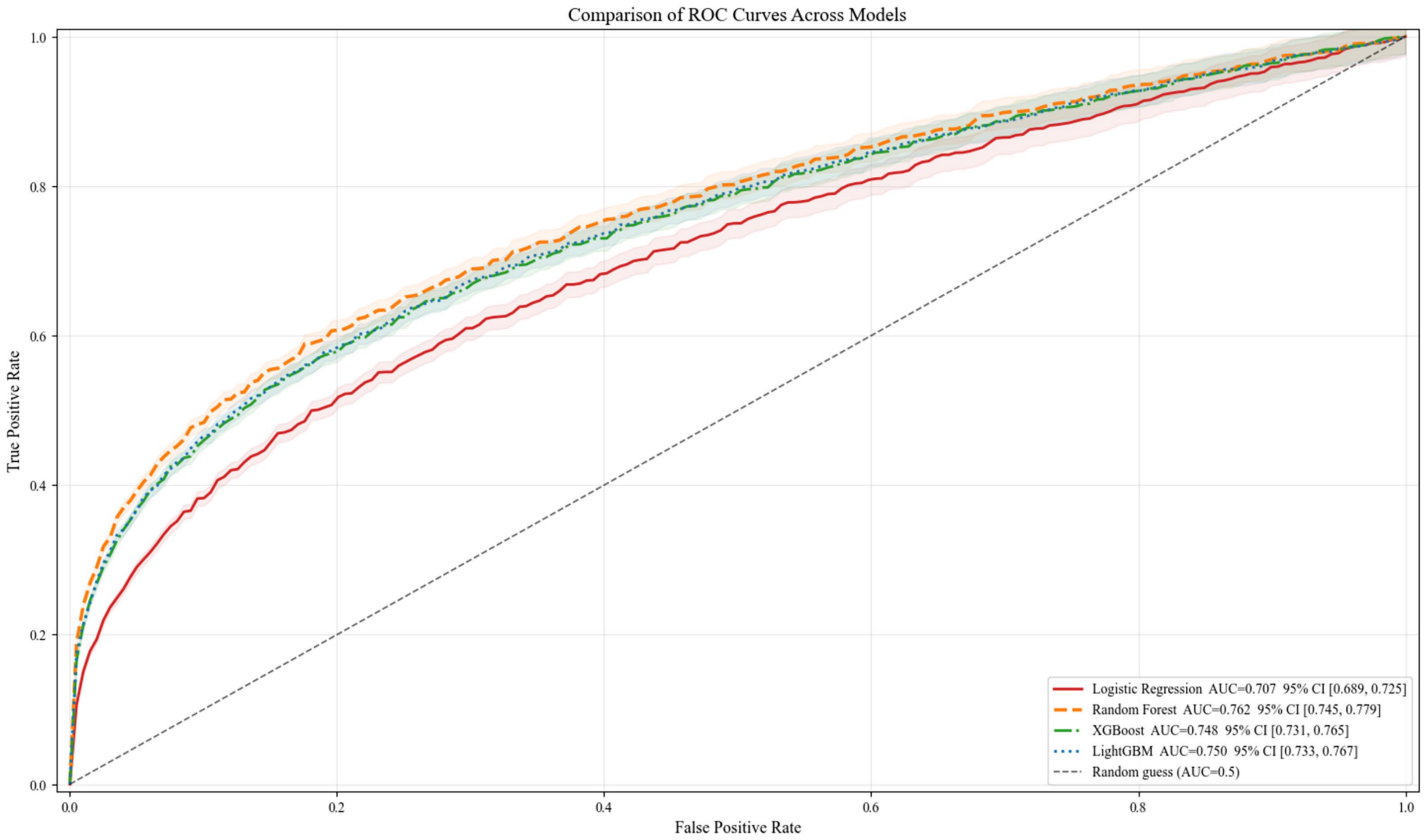
ROC curve plot.

The grid parameter tuning results in Table 2 demonstrate that RF achieves an AUC of 0.762, Accury of 0.704, and F1 score of 0.696 under the combination of ‘n_estimators 100, max_depth 4, minimum_samples_split 4, minimum_samples_leaf 2, max_leaf_nodes 6, and min impurity_decrease 0’. These metrics surpass those of LightGBM (0.750, 0.703, 0.695), XGBoost (0.748, 0.701, 0.693), and LR (0.707, 0.662, 0.656). Furthermore, RF exhibited the narrowest 95% confidence interval, indicating optimal stability. The ROC curves in Figure 1 further demonstrate that RF’s orange dashed line consistently occupies the highest position across the entire FPR range. Its AUC significantly outperforms the other three models, providing intuitive validation of RF’s superior generalisation capability and robustness when handling the multidimensional, potentially collinear data in this study.

**Table 2:** List of parameter optimization results for four machine learning model algorithms.

Model name	Hyperparameter research space	Best combination	Accuracy	Precision	Recall	F1 Score	AUC
LR	C: [0.01, 0.1, 1, 10, 100]	1	0.662	0.668	0.645	0.656	0.707
	penalty: [l1, l2, elasticnet]	l2					
	solver: [liblinear, saga]	saga					
	max_iter: [100, 200, 500]	100					
	class_weight: [none, balanced]	none					
LightGBM	num_leaves: [16, 31, 64]	31	0.703	0.709	0.688	0.695	0.750
	max_depth: [3, 4, 5]	4					
	learning_rate: [0.05, 0.1, 0.2]	0.1					
	colsample_bytree: [0.8, 0.9, 1.0]	0.9					
	min_child_samples: [5, 10, 20]	10					
XGBoost	n_estimators: [50, 100, 200, 300]	200	0.701	0.708	0.686	0.693	0.748
	max_depth: [2, 4, 6, 8]	4					
	learning_rate: [0.05, 0.1, 0.2]	0.1					
	subsample: [0.8, 1.0, 1.2]	0.8					
	gamma: [0, 0.1, 0.2]	0					
	reg_lambda: [0, 1, 5]	1					
RF	n_estimators: [50, 100, 200, 300]	100	**0.704**	**0.711**	**0.689**	**0.696**	**0.762**
	max_depth: [2, 4, 6, 8]	4					
	min_samples_split: [2, 4, 6, 8]	4					
	min_samples_leaf: [1, 2, 3]	2					
	max_leaf_nodes: [4, 6, 8, 10]	6					
	min_impurity_decrease: [0, 0.01, 0.02]	0					

The meaning of the bold values is the optimized parameter values of the best model.

### Analysis of characteristic importance

**Figure 2:**
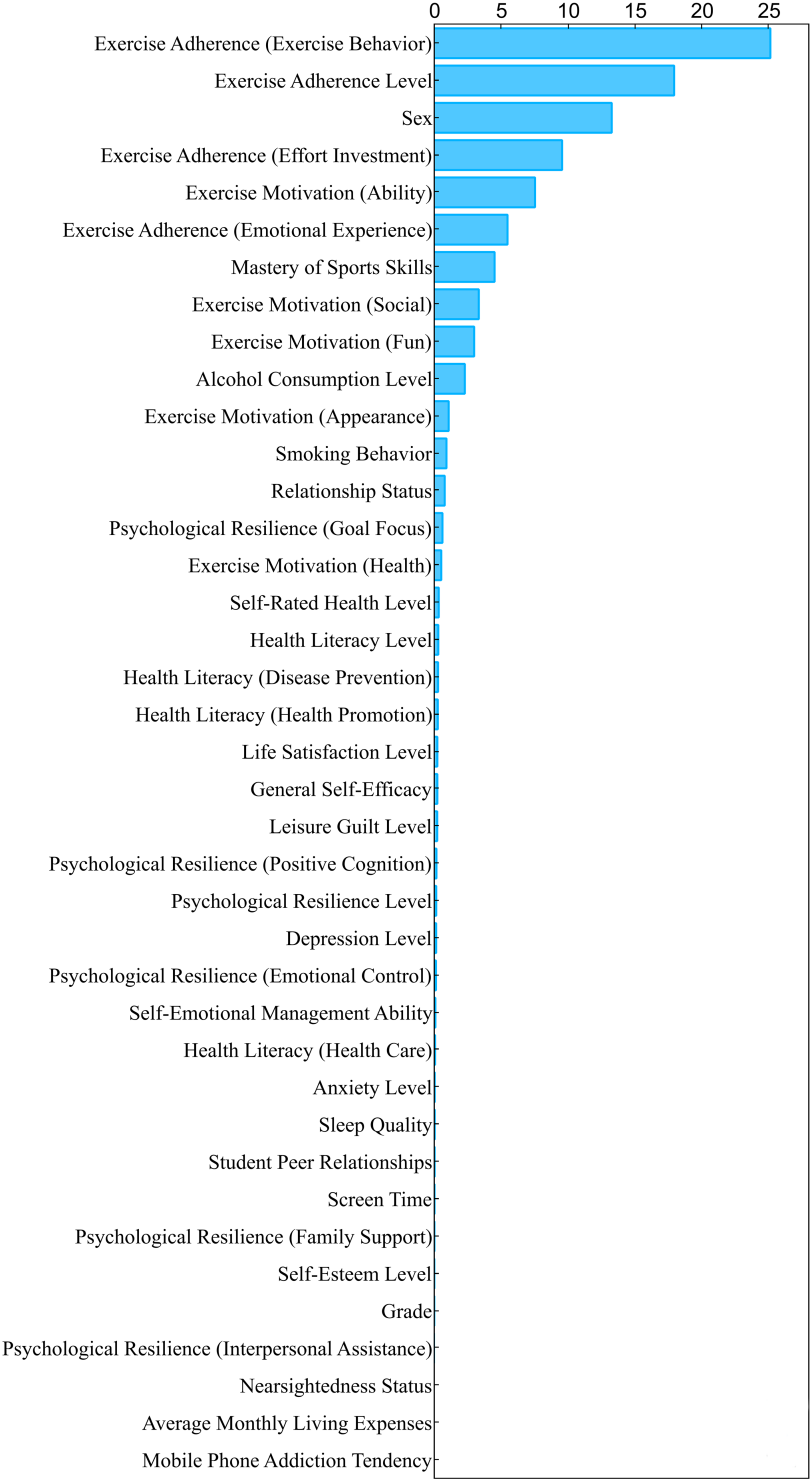
Feature contribution plot.

Figure 2, the Feature Contribution Plot, illustrates the characteristic importance of each feature within the model. A higher feature contribution indicates a more crucial role in the model’s predictive performance. Results indicate that exercise adherence (exercise behaviour), exercise adherence level, sex, exercise adherence (effort investment), exercise motivation (ability), exercise adherence (emotional experience), mastery of sports skills, exercise motivation (social), exercise motivation (fun), and alcohol consumption level rank among the top 10 contributing variables.

**Figure 3:**
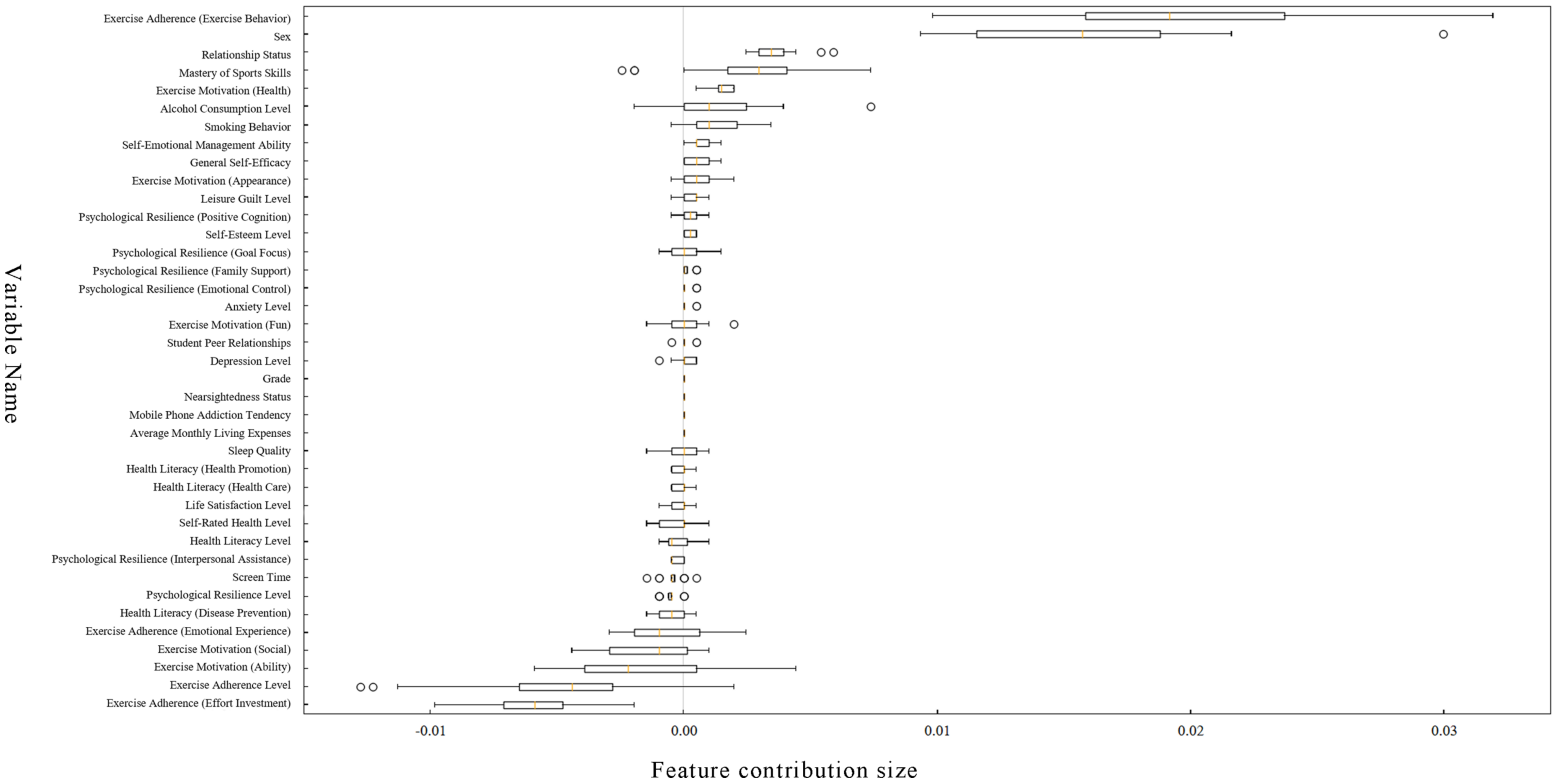
Feature contribution chart for permuted variables.

Figure 3 is a Feature Contribution Chart for Permuted Variables. This chart evaluates the importance of a feature by randomly permuting the values of each feature and observing the change in model performance. Results indicate that variables with the highest positive contribution values are: exercise adherence (exercise behaviour), sex, relationship status, mastery of sports skills, exercise motivation (health), alcohol consumption level, and smoking behaviour. Variables with the highest negative contribution values are: exercise adherence [exercise adherence (emotional experience), exercise motivation (social), exercise motivation (ability), exercise adherence level, and exercise adherence (effort investment)].

The slight discrepancies in feature ranking between Figures 2, 3 stem from the differing principles underlying these two evaluation methods. Figure 2 reflects a feature’s overall capability to separate nodes during model construction, whereas Figure 3 measures the feature’s independent contribution to the model’s predictive performance. For instance, the composite variable “exercise adherence level” ranks highly in Figure 2 because it correlates strongly with multiple sub-dimension variables and is frequently utilised during decision tree construction. However, in Figure 3‘s permutational testing, its information is overshadowed by its sub-dimensions, diminishing its independent contribution. Conversely, the sex variable, owing to its lower correlations with other variables, exhibits a more pure manifestation of its independent predictive capability in the permutational importance test, hence achieving a higher ranking. These discrepancies are common phenomena in RF analyses, collectively validating the importance of core factors from multiple perspectives.

**Figure 4:**
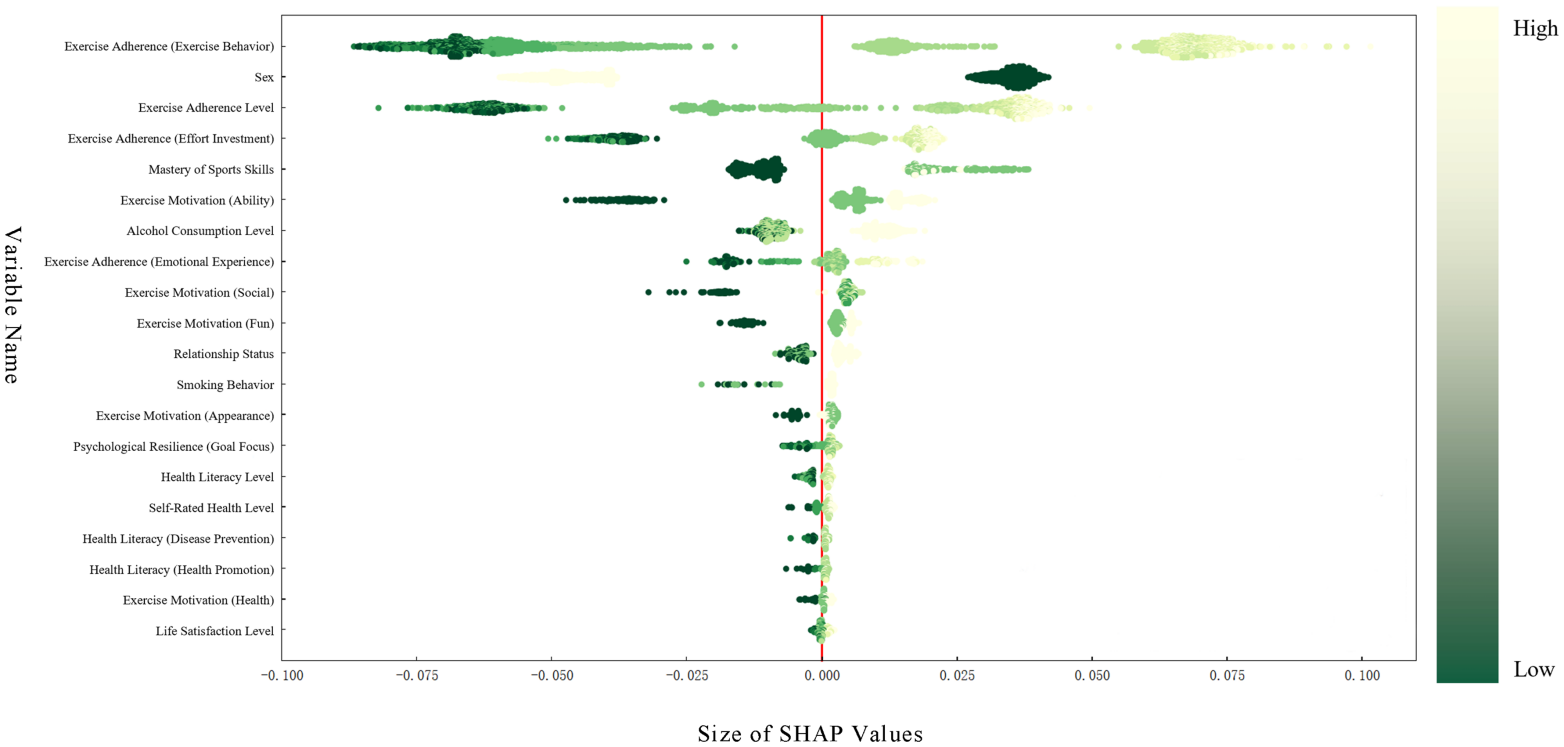
Schematic diagram of SHAP.

Figure 4 presents a summary diagram of SHAP (SHapley Additive exPlanations), wherein dark green points denote smaller values of the feature variable. Should these points exhibit negative SHAP values, it indicates that low-value features exert a negative influence on the dependent variable; conversely, positive SHAP values suggest a positive effect. Light green points represent the opposite scenario. The results in Figure 4 indicate that the top 10 contributing variables are: exercise adherence (exercise behaviour), sex, exercise adherence level, exercise adherence (effort investment), mastery of sports skills, exercise motivation (ability), alcohol consumption level, exercise adherence (emotional experience), exercise motivation (social), and exercise motivation (fun) for exercise adherence. SHAP analysis reveals that high exercise behaviour levels, being male, greater mastery of sports skills, moderate alcohol consumption, and high identification with the ‘exercise motivation(ability)’ category all significantly increase the likelihood of meeting PA standards. Conversely, negative SHAP values associated with being female and abstaining from alcohol reduce the probability of achieving these standards.

## Discussion

This study systematically identified key predictors of PA among university students through characteristic importance analysis of a RF model. It must be emphasised that, owing to the inherent limitations of cross-sectional research designs, the findings reveal statistical associations and predictive significance between variables without enabling inferences of strict causality. Nevertheless, exercise adherence and its sub-dimensions, exercise motivation, sex, mastery of sports skills, and alcohol consumption level, were confirmed as the most critical predictors. These findings align with prior literature while deepening our understanding of PA among university students.

The findings reveal that exercise adherence is the most significant predictor of PA, consistent with prior research (61). However, this study further illuminates the independent contribution of exercise adherence’s sub-dimensions to PA, an aspect that has been seldom addressed in previous investigations. Among these, exercise adherence (exercise behaviour) stands as the most crucial predictor of PA. Descriptive analysis indicates that university students who consistently achieved the target PA level scored higher on exercise adherence (exercise behaviour) than those who failed to meet the standard. The SHAP summary plot from the RF model also shows that exercise adherence (exercise behaviour) values are distributed widely and ranked prominently, underscoring its significant role in facilitating the attainment of PA benchmarks. In this study, exercise adherence represents the individual level within the SEM. Active participation in physical exercise yields multiple benefits: psychologically, it enhances mental health and well-being, while alleviating depression, stress, and anxiety (44); physiologically, it improves cardiorespiratory fitness, increases muscle mass and strength, and enhances learning and memory capabilities (62). Current research indicates that a key challenge in promoting PA lies in the discrepancy between individual exercise intentions and actual behaviour—the intention-behaviour gap. Despite strong intentions to exercise, individuals often fail to implement corresponding behaviours due to various influencing factors (63). In this study, the high SHAP value for exercise adherence (emotional experience) at the individual level, alongside the significant contribution of exercise motivation (social) at the interpersonal-organisational level to the predictive model, reveals the crucial role of emotional experiences and social motivation in promoting exercise behaviour. According to the concept of self-efficacy within social cognitive theory (64), when individuals exercise, beyond the act of adherence itself, the ‘emotional value’ derived from this behaviour may also play a role. This ‘emotional value’ may include, but is not limited to, encouragement and praise from those around them, and the social attributes gained from finding friends to exercise with consistently (65). Previous research corroborates this, as evidenced by a 2021 study that demonstrated the significant influence of enjoyment and motivation on exercise adherence (66). Another study showed that perceived self-efficacy among exercisers positively impacts the fulfilment of fundamental psychological needs (67). Therefore, to promote exercise adherence among university students, enhance PA levels, and bridge the gap between exercise intention and behaviour, intervention strategies should be designed across different levels of the SEM. At the individual level, psychological counselling and emotional management training should be employed to strengthen students’ self-efficacy and motivation for exercise. At the interpersonal level, social mechanisms such as sports groups, physical education clubs, and recreational sports events should be utilised to enhance peer support and social motivation, thereby transforming positive emotional experiences into sustained exercise behaviour.

Sex emerged as the second most significant predictor of PA among university students. SHAP analysis revealed positive SHAP values for males and negative values for females, confirming that males were significantly more likely than females to meet PA standards (63.8% vs 28.9%), consistent with prior research findings (68). Although sex itself, as a physiological characteristic, is not amenable to intervention, the socio-cultural preferences and behavioural patterns it reflects warrant attention. Existing research demonstrates an inequality in PA between boys and girls, with girls typically engaging in less PA than boys (69, 70). This disparity is more pronounced in high-income countries and those with higher Human Development Index rankings (71). Further research indicates that sex disparities narrow during vigorous-intensity PA but widen during moderate-intensity PA (72). This may stem from girls’ greater participation in aerobic exercises of lower intensity (56), coupled with perceived deficiencies in physical stamina and motivation for athletic pursuits (73). Concurrently, this may relate to sex-role socialisation processes. Male tend to perceive PA as a means of demonstrating strength and competitiveness, making them more likely to choose challenging, high-intensity activities to assert their masculinity, thereby achieving higher levels of PA. Female conversely, may prioritise the social aspects of sport or appearance enhancement. A toolkit document on sex equality in sport, jointly developed by the European Union and the Council of Europe, further indicates that men are more likely to engage in sport or PA for recreation (33%), socialising with friends (22%), or enhancing physical performance (29%). Female, conversely, are more concerned with weight management (24%), improving appearance (21%), or counteracting the effects of ageing (15%) (74). Consequently, interventions addressing female university students’ sporting challenges may be implemented at both the organisational and policy levels within SEM. At the organisational level, improvements can be made by restructuring physical education curricula, offering diverse intensity levels and interest-based options, and enhancing campus sports environments and institutional arrangements. At the policy level, integrating sex equality metrics into campus sports assessment and management systems encourages institutions to foster sex-friendly sporting environments. Such interventions can diminish the singular focus on competitive outcomes, emphasising instead health, aesthetic appreciation, and social benefits, thereby increasing female students’ motivation and participation in PA.

The findings of this study further indicate that exercise motivation (ability), specifically ‘I exercise to improve my athletic skills,’ and the mastery of sports skills are also significant predictors of PA among university students. SHAP analysis revealed that both a high level of identification with competence-based exercise motivation and the mastery of more sports skills corresponded with positive SHAP values. Descriptive analysis showed that the rate of meeting PA standards was significantly higher among students who mastered two or more skills (52.4%) compared to those who mastered fewer skills (zero skills: 22.6%; one skill: 30.6%). Research indicates that the development of sports skill competence is a primary underlying mechanism promoting individual participation in PA (75), with greater mastery of sports skills facilitating increased engagement in PA. A long-term randomised controlled trial similarly found that students in the specialised sports skills training group demonstrated significantly superior PA levels and physical fitness compared to the general physical education class group (76). This may be achieved through two pathways: the self-efficacy pathway and the social support pathway. As suggested by self-efficacy theory (64) and social cognitive theory (77), university students’ PA behaviour is influenced not only by their own cognitive factors but also by their surrounding environment. When individuals master more sports skills, they are more likely to perform well in PA, which further enhances their willingness to participate and thereby increases their PA levels. Concurrently, students proficient in multiple sports skills are more likely to engage in diverse activities, such as combining endurance and strength training (62), thereby increasing their overall PA levels. In certain team sports, activities requiring cooperation (such as basketball or volleyball) can better promote communication and interaction among college students, enhance the fun of physical activity and social attributes (78, 79). Within the SEM framework, these interventions operate at both individual and organisational levels. At the individual level, self-efficacy and body confidence can be enhanced through skill training and feedback mechanisms. At the organisational level, institutional support, including curriculum design, sports resource allocation, and teacher guidance, provides students with a sustained environment for skill development. The synergistic effect of these approaches fosters a positive cycle, progressing from ‘capability enhancement’ to ‘behavioural adherence,’ thereby significantly elevating PA levels.

Beyond exercise adherence, the exercise motivation, and mastery of sports skills, this study also identified complex predictive role of alcohol consumption level on PA. SHAP analysis revealed moderate drinking was associated with positive SHAP values, whereas abstinence was linked to negative SHAP values. Descriptive data further indicated a higher proportion of ‘occasional drinkers’ in the meeting-the-guidelines group compared to the non-meeting-the-guidelines group (49.8% vs 50.2%). Overwhelming evidence shows that alcohol consumption and smoking inflict damage upon the body (80–85). Chronic alcohol consumption leads to alcohol dependence, a rewarding, chronic, recurrent disorder causing significant health harm (86) to the nervous system, liver, digestive system, immune system, and cardiovascular system (80). Smoking causes multiple fatal diseases, including lung Cancer, respiratory diseases, and cardiovascular diseases (such as coronary heart disease) (87). A cross-sectional study revealed synergistic effects between smoking and alcohol consumption, with both substances jointly damaging the liver (88). Consequently, reducing alcohol intake and smoking is crucial for maintaining university students’ physical health, while engaging in PA offers a potential solution to this issue. One study demonstrated a linear inverse relationship between PA levels and alcohol consumption: individuals who engage in more frequent PA consume less alcohol, whereas those with lower PA levels tend to consume relatively more (89). Another study indicates that individuals with higher levels of PA are less likely to smoke (90). These findings align with the present study, potentially suggesting that moderate PA may reduce excessive alcohol consumption level, though the complex relationship between the two warrants further investigation. Concurrently, regular moderate-to-vigorous physical exercise can counteract the adverse metabolic effects of alcohol consumption on liver function, inflammation, and lipid profiles (89). A potential mechanism may be that PA modulates reward systems and emotional states, thereby reducing alcohol intake driven by its rewarding properties and partially counteracting its adverse effects, thereby decreasing consumption (91). However, it should be noted that other studies have demonstrated a positive correlation between PA and alcohol consumption, with both intensity and duration of PA increasing alongside alcohol intake (92, 93). This aligns with the findings of the present study. A plausible explanation may be that moderate drinkers exhibit greater social engagement and extroverted traits, thereby facilitating participation in collective sporting activities. Some research has also indicated that social drinking may to some extent reflect an individual’s social activity levels and sense of group belonging (94), which corresponds with the social support mechanisms at the interpersonal level within the SEM. However, it is essential to distinguish the behavioural engagement effects of this ‘social drinking’ from the health risks associated with ‘physiological drinking’: the former may temporarily enhance social motivation and exercise participation, while the latter continues to increase negative health outcomes such as liver damage and metabolic disorders over the long term (95). Concurrently, negative SHAP values among abstainers do not imply abstinence itself is harmful. Rather, they may reflect social avoidance or psychologically conservative traits among some individuals in this group, indirectly reducing their opportunities for sports participation. This interpretation is supported by psychosocial research indicating that individuals with low social motivation or avoidant personality tendencies are more likely to exhibit concurrently low PA levels (96). This overlapping neurochemical effect may constitute a biological basis for the positive correlation between PA and alcohol consumption (97). From a SEM perspective, multi-level health promotion strategies can be designed to address drinking behaviour. At the individual level, health education and self-control training should be reinforced; at the interpersonal level, peer role models and social support can influence drinking behaviour; at the organisational level, campus health behaviour management systems should be improved; and at the community and policy levels, public health advocacy and institutional restrictions should be promoted. This approach fosters a supportive environment conducive to PA and healthy behaviour across multiple ecological systems.

The strength of this study lies in its analysis based on large-sample cross-sectional survey data, which innovatively employs machine learning techniques to elucidate the predictive mechanisms of PA among university students. Compared to traditional statistical methods, this approach more effectively captures the nonlinear relationships and interactions between variables. Integrating the SEM framework, the study systematically synthesised multidimensional measurement indicators encompassing personal characteristics, interpersonal interactions, and organisational environments. This established a multi-level predictive factor analysis system, providing multidimensional evidence to support the formulation of precise health intervention strategies. However, several research limitations should be noted. Firstly, the cross-sectional design imposes methodological constraints on revealing causal relationships between variables and capturing temporal dynamic evolution. These findings only indicate associations or predictions of factors. Secondly, the model accuracy in this study was approximately 70%, potentially attributable to: (1) measurement error in PA assessment: the use of a self-reported questionnaire (PARS-3) may introduce recall bias and social desirability bias, leading to inherent inaccuracies compared to objective measurement tools such as accelerometers; (2) Categorical imbalance: although the proportion of the meeting-the-standard group (44.5%) versus the not-meeting-the-standard group (55.5%) was not extremely skewed, this imbalance may still pose a minor challenge to the model’s ability to learn patterns in the minority category; (3) Unmeasured SEM’s factors: Given the study’s scope, the model primarily incorporated individual and interpersonal-organisational level variables, failing to encompass critical community and policy-level factors such as accessibility of campus sports facilities, curriculum design, and sports scholarship policies. This omission of built and policy environments may limit the model’s overall explanatory power and the systematic nature of intervention measures; (4) Sample and feature limitations: Despite the substantial overall sample size, certain subgroups exhibited relatively small sample sizes, and feature engineering may not have fully captured all complex nonlinear relationships predicting PA.

Future research may deepen exploration in this field through the following avenues: Firstly, employing longitudinal tracking designs and causal inference models to clarify causal pathways and temporal dynamics between predictive factors and PA. Secondly, constructing multi-level models integrating community and policy dimensions, systematically incorporating macro-level variables such as campus spatial environments, accessibility of sports resources, and local health policies to address the hierarchical gaps in the SEM used herein. Third, actively promote multi-source data integration. Beyond self-reported data, combine objective behavioural data such as accelerometer readings, smartphone sensor data, and campus card transaction records to capture PA patterns and contexts more precisely and comprehensively, thereby effectively reducing measurement errors inherent in single self-reporting sources. Fourthly, explore more advanced machine learning techniques to handle complex interaction effects, employing strategies such as oversampling to further optimise the model’s classification performance across categories. Through these measures, we aim to comprehensively elucidate the predictive mechanisms of PA among university students, thereby providing stronger evidence-based support for developing precise intervention strategies.

## Conclusion

This study systematically identified key factors predicting PA levels among Chinese university students using a RF model. Exercise adherence and exercise motivation ranked highly within the model. Consequently, to effectively enhance students’ PA levels, interventions should prioritise improving exercise adherence, with particular emphasis on strengthening the “emotional value” and social attributes of PA. Designing engaging and socially interactive PA can effectively satisfy students’ intrinsic psychological needs, bridging the gap between exercise intent and actual behaviour. Concurrently, SHAP values clearly indicate that female identity correlates with lower attainment probabilities, necessitating targeted attention to female students’ barriers to PA participation. Interventions should strive to create more female-friendly sporting environments, de-emphasising purely competitive orientations in favour of highlighting the health, aesthetic, and social benefits of participation. Systematically training students in multiple sports skills represents another effective pathway. This recommendation is supported by two highly ranked factors: mastery of sports skills and ability-oriented exercise motivation. Enhancing sports skills not only directly boosts personal efficacy but also lays the foundation for engaging in diverse PA. Future health promotion efforts should closely align with these key predictive factors, designing multi-tiered, precision-targeted intervention strategies to effectively elevate PA levels among Chinese university students through data-driven approaches.

## Data Availability

The raw data supporting the conclusions of this article will be made available by the authors, without undue reservation.
